# Machine Learning Algorithms for Processing and Classifying Unsegmented Phonocardiographic Signals: An Efficient Edge Computing Solution Suitable for Wearable Devices

**DOI:** 10.3390/s24123853

**Published:** 2024-06-14

**Authors:** Roberto De Fazio, Lorenzo Spongano, Massimo De Vittorio, Luigi Patrono, Paolo Visconti

**Affiliations:** 1 Department of Innovation Engineering, University of Salento, 73100 Lecce, Italy; roberto.defazio@unisalento.it (R.D.F.); lorenzo.spongano@unisalento.it (L.S.); massimo.devittorio@unisalento.it (M.D.V.); luigi.patrono@unisalento.it (L.P.); 2 Center for Biomolecular Nanotechnologies, Italian Institute of Technology, 73010 Arnesano, Italy

**Keywords:** classification, unsegmented phonocardiogram, machine learning, binary classifier, multiclass classifier

## Abstract

The phonocardiogram (PCG) can be used as an affordable way to monitor heart conditions. This study proposes the training and testing of several classifiers based on SVMs (support vector machines), k-NN (k-Nearest Neighbor), and NNs (neural networks) to perform binary (“Normal”/”Pathologic”) and multiclass (“Normal”, “CAD” (coronary artery disease), “MVP” (mitral valve prolapse), and “Benign” (benign murmurs)) classification of PCG signals, without heart sound segmentation algorithms. Two datasets of 482 and 826 PCG signals from the Physionet/CinC 2016 dataset are used to train the binary and multiclass classifiers, respectively. Each PCG signal is pre-processed, with spike removal, denoising, filtering, and normalization; afterward, it is divided into 5 s frames with a 1 s shift. Subsequently, a feature set is extracted from each frame to train and test the binary and multiclass classifiers. Concerning the binary classification, the trained classifiers yielded accuracies ranging from 92.4 to 98.7% on the test set, with memory occupations from 92.7 kB to 11.1 MB. Regarding the multiclass classification, the trained classifiers achieved accuracies spanning from 95.3 to 98.6% on the test set, occupying a memory portion from 233 kB to 14.1 MB. The NNs trained and tested in this work offer the best trade-off between performance and memory occupation, whereas the trained k-NN models obtained the best performance at the cost of large memory occupation (up to 14.1 MB). The classifiers’ performance slightly depends on the signal quality, since a denoising step is performed during pre-processing. To this end, the signal-to-noise ratio (SNR) was acquired before and after the denoising, indicating an improvement between 15 and 30 dB. The trained and tested models occupy relatively little memory, enabling their implementation in resource-limited systems.

## 1. Introduction

Traditional non-invasive diagnostic tools for cardiovascular diseases often involve expensive equipment and specialized medical personnel, limiting their use to specialized clinics and hospitals [1]. The phonocardiogram (PCG), in which the cardiac sound signals are used to analyze the heart’s health, offers an affordable alternative to the traditional non-invasive diagnostic tools. Various heart conditions, such as valvular heart disease, congenital heart disease, heart failure, high blood pressure, and coronary artery diseases (CAD), can be detected via the auscultation and interpretation of heart sounds [2]. The typical process of PCG signal classification involves denoising, segmentation, and classification. It is worth observing that PCG signals are susceptible to various noise sources, which can hamper proper signal segmentation and classification [3,4].

The heart sound segmentation consists of identifying the fundamental heart sounds (FHS), “S1” and “S2”, and additional sounds such as “S3”, “S4”, murmurs, and clicks, which might be present but are harder to identify and often indicate pathological conditions [5,6]. “S1” occurs at the systole start, whereas “S2” occurs at the beginning of the diastole. Time–frequency analysis is crucial for an in-depth analysis of PCG signals because they are not stationary. Some ways to perform this time–frequency analysis include short-time Fourier transform (STFT) and wavelet transform (WT) [6,7].

Several studies in the literature developed PCG binary classifiers to classify normal and pathological PCG signals. P. Langley and A. Murray developed a threshold classifier for the wavelet entropy without employing any segmentation or signal processing algorithms using the Physionet/CinC 2016 dataset as the reference data [8]. This classifier achieved a balanced accuracy equal to 77% (98% sensitivity, 58% specificity). Similarly, in a successive study, P. Langley and A. Murray classified normal and pathological PCG signals in the 2016 Physionet/CinC Challenge dataset using MATLAB without heart sound segmentation algorithms [9]. At first, the authors used threshold classifiers for the normalized spectral amplitude and the wavelet entropy, achieving 70% (65% specificity, 75% sensitivity) and 76% balanced accuracy (54% specificity, 98% sensitivity), respectively. Then, the analysis was repeated using minimal noise signal segments. In addition to the threshold classifiers for each of the selected features, the authors utilized a classification tree utilizing all features simultaneously, yielding a 79% balanced accuracy (80% specificity, 77% sensitivity). The classification of normal and pathological PCG signals without segmentation algorithms was also performed by P. Krishnan et al., who developed a one-dimensional convolutional neural network (CNN) utilizing 1081 PCG recordings as the reference data [10]. This classifier achieves an overall accuracy equal to 85.65% and an 85.74% balanced accuracy (86.73% sensitivity, 84.75% specificity). Similarly, N.E. Singh-Miller et al. developed a method to discern normal and pathological PCG signals using spectral features without explicitly segmenting the “S1” and “S2” sounds. The authors employed a random forest (RF) regressor for classification, which achieved an 81% balanced accuracy (76% sensitivity, 87% specificity) [11].

However, PCG segmentation algorithms enable the extraction of further PCG features useful for normal/pathological heart sound classification. Hence, several studies in the literature used segmentation algorithms to detect the “S1” and “S2” sounds. M.A. Goda and P. Hajas utilized a support vector machine (SVM)-based classifier to differentiate between normal and pathological PCG signals from the Physionet/CinC 2016 dataset (in particular, 1000 samples with a 1:1 ratio between normal and pathological signals) [12]. This classifier obtained an 81.2% balanced accuracy (85.2% specificity, 77.2% sensitivity). In a similar study, H. Tang et al. developed an SVM classifier to discern between normal and pathological PCG signals utilizing a feature set of 515 features from nine domains, including time interval, frequency spectrum, and entropy [13]. This classifier achieved an 88% balanced accuracy (88% sensitivity, 87% specificity). Other studies in the literature developed PCG classifiers based on machine learning (ML)/deep learning (DL) models with augmented complexity and employing segmentation algorithms. T. Nilanon et al. utilized the PhysioNet/CinC 2016 dataset to deploy a CNN-based binary model to classify 5-s segments of normal and pathological PCG signals; the classifier achieved an 81.1% balanced accuracy (77% sensitivity, 85.3% specificity) [14]. Similarly, M.N. Homsi and P. Warrick proposed an ensemble of 20 classifiers to classify normal and pathological segmented PCG signals from the Physionet/CinC 2016 dataset, focusing on outlier detection [15]. This method yielded a balanced accuracy of 80.1% on the test set (79.6% sensitivity, 80.6% specificity). Likewise, E. Kay and A. Agarwal proposed a classifier based on DropConnected neural networks (NNs) trained on time–frequency and inter-beat features, an algorithm for classifying segmented PCG signals from the Physionet/CinC 2016 dataset as normal or pathological [16]. This classifier achieves an 85.2% balanced accuracy on the test data. In a similar study, C. Potes et al. developed a model combining a feature-based classifier using AdaBoost and a deep learning-based classifier using a CNN trained and tested on the PhysioNet/CinC 2016 dataset, achieving an 86.02% balanced accuracy, 92.24% sensitivity, and 77.81% specificity [17]. T. Liu et al. developed an SVM-based classifier to discern between normal PCG signals and those with CAD, using heart sound segmentation algorithms, achieving a 90.9% accuracy (87.8% sensitivity, 93.0% specificity, 90.4% balanced accuracy) using a custom dataset with 991 samples [18]. The multiclass classification was performed by Y. Zeinali and S.T.A. Niaki in reference [19]. The authors classified PCG signals from the PASCAL dataset to detect the presence of the “S3” and “S4” sounds, each associated with specific cardiovascular diseases. The study employed various machine learning algorithms, such as SVMs, RF, and Gradient Boosting Classifier (GBC), to classify the sounds. The latter yielded an 87.5% overall accuracy. S. B. Shuvo et al. developed a convolutional recurrent neural network (CRNN)-based classifier to identify cardiovascular diseases using PCG signals, which requires 7.96 MB [20]. This model achieved a 99.6% accuracy, 99.6% precision, 99.5% sensitivity, and 99.6% F1-score on a PCG signal dataset available on GitHub. In contrast, it yielded 86.6% accuracy, 93.3% precision, 99.5% sensitivity, and 99.6% F1-score on the Physionet/CinC 2016 dataset related to the binary classification of normal and pathological signals. Lastly, N. Baghel et al. developed a CNN-based classifier that uses heart sounds to diagnose cardiac disorders automatically, yielding a 98.6% accuracy using 2000 samples from data available on GitHub [21].

This paper presents the development of binary (Normal/Pathological) and multiclass PCG classifiers to rapidly and massively detect and discern valvular heart diseases. The latter discerns between various kinds of heart diseases, namely mitral valve prolapse (MVP), CAD, and benign murmurs. Different ML/DL models, like SVMs, neural networks (NNs), or *k*-Nearest Neighbor (k-NN), are trained and tested using datasets created from the PhysioNet/CinC 2016, which contains recordings labeled by expert medical staff of both normal and pathological PCG signals; in particular, two datasets were created containing 482 (241 normal and 241 pathological) and 826 (287 normal, 287 CAD, 134 MVP, 118 murmurs) signals used for training and testing the binary and multiclass classifiers. These signals are pre-processed by removing spikes, reducing noise, and normalization; then, they are split into 5 s frames using a moving window with a 1 s shift, enabling augmenting the number of observations used to train and test the classifiers. In this way, binary and multiclass datasets, including 10104 and 13136 frames, respectively, are gathered, randomly removing excess frames from each class, thus balancing the two datasets. Subsequently, a set of features in various domains (i.e., time, frequency, statistical, wavelet, and MFCC (Mel Frequency Cepstral Coefficient) domains) are extracted from each PCG frame and used to train and test the classifiers. The resulting datasets are used to train and test different binary and multiclass classifiers via the MATLAB Classification Learner application. The test results demonstrated that k-NN classifiers obtain the best performance for both binary and multiclass classification, reaching 98.7% accuracy and F1-score. Nevertheless, these last classifiers feature a relatively high memory occupation (up to 14.1 MB for multiclass classification). Instead, NNs are the best trade-off between performance (up to 96.0% accuracy and F1-score) and memory requirements (up to 735 kB). The resulting classifiers are suitable to be implemented in a resource-limited programmable system. The main strengths of the proposed work include the following:

- The pre-processing chain of PCG signals reduces noise and normalizes signals, enabling performance not dependent on signal quality, making the classifiers practical for real-world scenarios;
- No segmentation algorithms are employed in the feature engineering phase to identify the heart sounds, such as “S1” and “S2”, since these algorithms are computationally demanding and typically require additional information that cannot be derived directly from PCG signals [22,23];
- Lightweight ML/DL models (e.g., SVMs, NNs, and k-NN) are considered, enabling their implementation on resource-limited platforms [24];
- Only scalar features were selected to further limit the classifiers’ complexity;
- The models occupy a small amount of memory, enabling the classifiers’ implementation on memory-limited platforms, making them suitable for edge computing-based wearable devices or mobile healthcare applications, allowing for real-time monitoring and diagnostics [1];
- Both binary and multiclass classification are performed, highlighting the classifier’s versatility in different diagnostic scenarios.

The remainder of this article is organized as follows: Section 2 describes various datasets containing PCG signals and the code developed in MATLAB R2021b. Section 3 highlights the main results of this research work, whereas Section 4 discusses the results mentioned above. Lastly, Section 5 analyzes the strengths and main findings of the work.

## 2. Materials and Methods

### 2.1. Analysis of Open-Access PCG Signal Datasets

Below, the main open-access datasets containing PCG signals are described, namely the PASCAL, the 2011 Catania Heart Sounds, and the Physionet/CinC 2016 datasets. The criteria adopted for the dataset selection include the number and duration of signals in the dataset and the presence of labeling of PCG signals by expert medical personnel, allowing for the development of supervised classifiers.

The PASCAL (Pascal Classifying Heart Sound Challenge) dataset contains 859 PCG recordings gathered from both the iStethoscope iOS application and hospital clinical trials using a digital stethoscope, with durations ranging from 1 to 30 s [25]. The dataset is divided into two sets: A and B. The first set consists of 176 heart sounds (23.58 min of recordings) labeled into the categories “normal”, “breath”, “extra heart sound”, and “artifact”; the second set contains 656 signals (a total of 71.64 min) labeled into three categories: “normal”, “murmur”, and “extrasystole”.

Another widely used dataset is the 2011 Catania Heart Sounds (CTHS) dataset, which comprises 412 PCG recordings acquired from 206 individuals while they were resting, using a ThinkLabs Rhythm electronic stethoscope, with a sampling rate of 11.025 kHz and 16-bit resolution [26]. However, this dataset does not provide diagnoses of heart diseases. A dataset that encompasses diagnoses of heart diseases is the PhysioNet/CinC 2016; this dataset comprises recordings of heart sounds collected from both healthy subjects and those with pathological conditions, such as MVP, mitral regurgitation (MR), aortic regurgitation (AR), aortic stenosis (AS), or CAD. The recordings were collected in various environments using clinical and nonclinical equipment, ranging in duration from a few seconds to several minutes, and are divided into two subsets: a training set and a test set [27,28]. All PCG signals in the dataset were resampled using a 2 kHz sampling rate and an antialiasing filter and then converted to the .wav format. It is important to note that the dataset is unbalanced, with a higher number of recordings classified as “normal” than as pathological. The training set comprises six databases (labeled “training-“ followed by the alphabet letters “a” to “f”) that contain a total of 3240 PCG signals acquired from 764 patients, with a duration between 5 and 120 s. Each database’s file name starts with the same letter, indicating the database to which it belongs, followed by a sequential random number. Also, each database includes a specific subset of the cardiovascular diseases mentioned above. Table 1 summarizes the contents of each database of the dataset’s training set.

The Physionet/CinC 2016 dataset was selected as the reference data for the classifiers trained and tested in this research because it contains the most signals among the analyzed datasets and diagnoses of the heart’s condition performed by expert medical personnel (Table 1). Specifically, only a portion of the Physionet/CinC 2016 training set is used for the binary and multiclass classifiers trained and tested in this work. Concerning the binary classification, 482 signals (241 normal and 241 pathological) are randomly selected from the “training-c”, “training-e”, and “training-f” sections. Similarly, for multiclass classification, 826 signals from the entire dataset are selected (normal (287), CAD (287), MVP (134), and benign murmurs (118)), including as many “normal” signals as the maximum number of signals among the pathologic classes. In both cases, nearly balanced datasets are created to optimize the training and testing of classifiers.

### 2.2. Pre-Processing, Feature Extraction, and Dataset Partition from the PCG Signals

**Figure 1:**
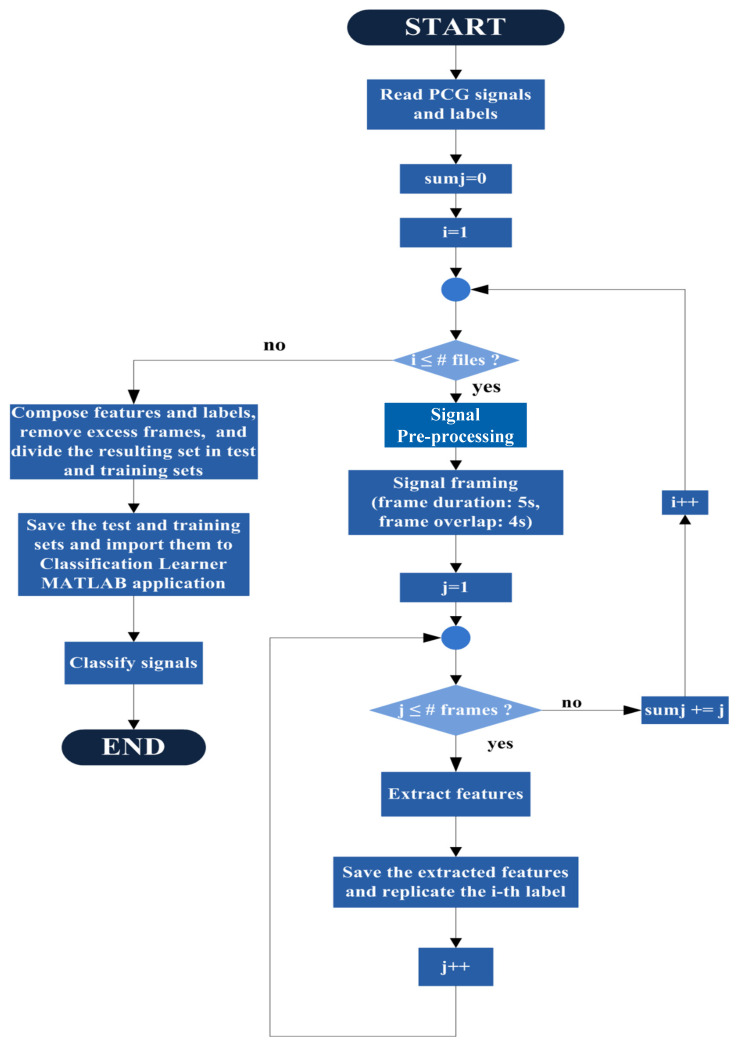
High-level flowchart for pre-processing, feature extraction, and dataset partition.

This work involves the training and the testing of different classifiers, namely SVMs, feed-forward NNs, and k-NN, to discern between normal and pathologic PCG signals (binary classification) and to detect various kinds of heart diseases, such as benign murmurs, coronary artery disease, or mitral valve prolapse (multiclass classification). MATLAB version 2021b (Mathworks Inc., Natick, MA, USA) was used to pre-process the PCG signals, extract features, and create the training and test datasets to be used in the Classification Learner application embedded in the MATLAB package for training and testing the different classifiers. Figure 1 depicts the high-level flowchart of the MATLAB code. The following steps are performed sequentially:

- PCG signals are read;
- For each PCG signal, the following tasks are performed:
  ○ Pre-processing to remove spurious spikes and reduce noise via a wavelet-based denoising algorithm. Then, the signal is filtered and normalized;
  ○ Division into 5 s frames with a 1 s shift. This operation enables the expansion of the size of the feature dataset and consequently reduces the over-fitting risk;
  ○ For each frame, the following tasks are carried out:
    ▪ Feature extraction. Thirty-three scalar features are extracted from multiple domains (i.e., time, frequency, statistical, wavelet, and MFCC domains). More details on the extracted features are detailed in Section 2.4;
    ▪ The extracted features are stored;
    ▪ Features belonging to the same signal are annotated;
  ○ The dataset obtained in the previous step is split according to the classes;
  ○ The excess frames are randomly removed according to the minimum number of frames across all classes;
  ○ Each class dataset obtained in the previous step is partitioned into training (80%) and test (20%) sets. In this way, the number of frames for each class is guaranteed to be equal for all classes;
  ○ Each training and test set is combined.

In addition to the methodology used to create the balanced training and test sets after the signals’ framing (with or without overlap), to consider a realistic scenario of using the trained models for classifying the PCG signal of a new patient under examination, a second strategy was implemented to create the training and test sets. First, the 241 normal and 241 pathological signals (total of 482 labeled signals) that make up the dataset were randomly distributed proportionally in the training and test sets (20% and 80%, respectively); then, the framing was carried out with a frame duration of 5 s and overlap of 0 or 4 s. In this way, differently from the methodology used previously, frames of the same signal all belong to the training or test set without the possibility that they can be included in both. After the framing step, the excess frames were randomly eliminated in both the training and test sets to obtain a perfect balance between normal and pathological frames. In summary, in the case of 0 s overlap in the framing step, the training set consists of 2012 frames (1006 normal and 1006 pathologic) and the test set of 504 frames (252 for both classes). In Section 3, the results related to the classifiers’ performance using the datasets thus obtained are reported and then compared with those achieved with the first method implemented to create the training and test sets.

Subsequently, the training and test datasets in “.xlsx” format were loaded into the MATLAB Classification Learner application. The training dataset was imported using the “cross-validation” methodology, which divided the dataset into 10 equal parts for validation. According to the previous description, all frames derived from a signal were marked with the same signal label after assessing that the entire signal shows the typical features related to the annotated pathology. This approach is justified, considering the cardiac pathologies evaluated are valvular; therefore, it is presumable that the dysfunction will recur in each cardiac cycle. Various machine learning models, including different types of SVMs, k-NN, and NNs, were trained using the Classification Learner application. After training, each model was tested using the test dataset obtained before. The datasets used to train and test the PCG classifiers have been attached in the Supplementary Materials (File S1).

### 2.3. PCG Signal Pre-Processing

As described in the previous section, the PCG signals were pre-processed to make them suitable for the following feature extraction; in detail, spike removal and denoising algorithms were applied to PCG signals. The spurious spike removal algorithm applied to the PCG signals is the one proposed by Schmidt et al. [22], and it is outlined as follows:

- The PCG signal is divided into 0.5 s frames;
- The maximum absolute amplitude (MAA) is determined in each frame;
- If at least one MAA is greater than thrice the median of all recorded MAAs, the following steps are initiated; otherwise, the spike removal algorithm ends:
  ○ Select the frame with the largest MAA;
  ○ Obtain the location of the MAA in the selected frame;
  ○ Define the beginning and the end of the spurious spike as the last zero-crossing point preceding the MAA and the first zero-crossing point after the MAA;
  ○ Set the points corresponding to the identified interval to zero;
  ○ Repeat until the spurious spikes are removed.

The denoising algorithm performs the wavelet decomposition up to a certain level and applies a threshold function for all the detail coefficients. Afterward, for each decomposition level, the approximation coefficients of the prior level are obtained by performing the inverse discrete wavelet transform using the current level’s approximation coefficients and detail coefficients with the application of the threshold function. This process is repeated until the signal is reconstructed with reduced noise. In particular, an optimization was performed regarding the optimal wavelet denoising parameters (wavelet typology, order, and level) (Table 2). The metric used to evaluate the different tested configurations is the average SNR between the input and output to the denoising block calculated on the entire dataset. According to the obtained results, the selected parameters for the denoising algorithm are as follows: fifth-order Daubechies’ wavelet (db5), universal threshold (i.e., $\sqrt{2\;ln(signal\;length)\;}$), level-dependent noise estimate, and soft thresholding.

After the denoising step, a fourth-order Butterworth bandpass filter with cutoff frequencies equal to 25 and 450 Hz is applied to emphasize the signal in the band of interest. In MATLAB, the filter is applied to the signal through the filtfilt function, which performs zero-phase filtering on the target signal. The combined use of the Butterworth filter and the denoising block enhances the performances in terms of overall accuracy in the binary classification compared to the case where such blocks are not used; this consideration is justified, as the signal-to-noise ratio increases by between 15 and 30 dB when the blocks are used, resulting in less noisy signals, as detailed further in Section 4. However, the Butterworth filter is not applied in the multiclass classifiers since they must also detect benign murmurs, which can have frequency components up to 1 kHz. The last pre-processing step consists of normalizing the filtered signal with respect to its maximum amplitude. Then, each signal is split into frames with a 5 s moving window with a 1 s shift, enabling the extension of the dataset. In detail, two balanced datasets constituted by 10,104 and 13,136 frames are created for binary and multiclass classification by randomly removing the excess frames for each class.

**Figure 2:**
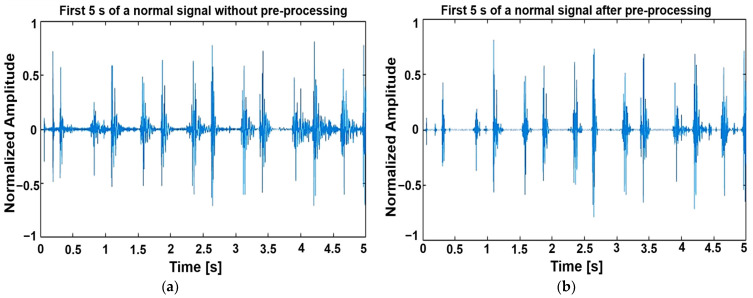

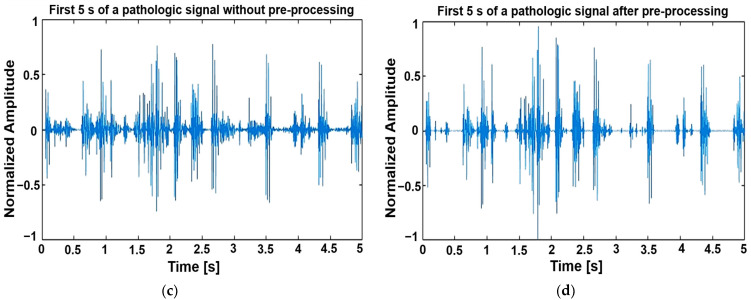
Normal and pathologic signals without and after pre-processing. (**a**) First 5 s of a normal signal without pre-processing. (**b**) First 5 s of a normal signal after pre-processing. (**c**,**d**): First 5 s of a pathologic signal without and after pre-processing.

Figure 2 shows an example of the first 5 s of normal and pathologic PCG signals before and after the pre-processing.

An analysis was carried out regarding the gender and age of the patients regarding the frame dataset generated for the binary classifiers. Out of 10,104 frames, 4779 frames (47.3%) are from women, while 5325 frames (52.7%) are from men. Furthermore, 30.5% of the total frames (3081 frames) are from patients aged less than or equal to 25 years; 16.0% are from patients aged between 26 and 50 (1620 frames), and 53.5% are from patients aged over 50.

### 2.4. Feature Selection and Extraction

Scalar features were chosen to reduce the classifiers’ complexity; furthermore, no segmentation algorithm to identify the “S1” and “S2” sounds was used to select the features since they are computationally demanding (e.g., the segmentation algorithms are often based on additional machine or deep learning models) and typically require other information that cannot be derived directly from the PCG signal, such as the R-peak’s duration, which is derived from the electrocardiogram (ECG) [23]. A feature set comprises 36 scalar features belonging to the time, frequency, statistical, wavelet, and MFCC domains. They were selected considering signal features, which carry significant information about the structure and characteristics of the signal, which may be sensitive to signal changes induced by cardiac pathologies. In particular, the wavelet entropies calculated on a 4-level decomposition were included among the selected features, allowing the signal’s complexity to be quantified in detail. These features can be particularly useful for diagnosing heart disease, where the complexity of the signal can provide clues to the presence of abnormalities. Using wavelet decomposition, it is possible to analyze the signal at various levels of detail, obtaining a complete view of its frequency and temporal characteristics [29]. Such features are extracted from each frame to constitute the dataset used to train and test the classifiers. The extracted features, their definitions, and their respective domains are summarized in Table 3.

### 2.5. Classifier Performance Evaluation Metrics

Regarding the classifiers considered, attention was paid to models suitable for implementation on embedded systems. In detail, SVMs, k-NN, and NNs are often used for embedded applications, given their simplicity, efficiency in classification, robustness, and versatility. Neural networks can guarantee excellent performance, quick and efficient inferences, and the ability to exploit models pre-trained on more powerful hardware and export them to embedded devices [31,32,33]. Furthermore, multiple optimized frameworks (such as TensorFlow Lite, ONNX Runtime, etc.) facilitate their implementation on embedded devices, making the most of the available hardware resources. Multiple configurations and settings were considered relative to the structures and parameters, as described in Section 3.

The metrics to evaluate the performance of the trained classifiers are the following: overall accuracy, sensitivity, specificity, precision, balanced accuracy, and F1-score. The true positives (TPs) are the correctly predicted positive classes, the false positives (FPs) are the incorrectly predicted positive classes, the true negatives (TNs) are the correctly predicted negative classes, and lastly, the false negatives (FNs) represent the incorrectly predicted negative classes. The overall accuracy (Acc) is obtained as in Equation (1):

(1) $$Acc=\frac{TP+TN}{TP+TN+FP+FN}$$

The sensitivity (Se), the specificity (Sp), and the precision (P) are obtained as in Equations (2)–(4):

(2) $$Se=\frac{TP}{TP+FN}$$

(3) $$Sp=\frac{TN}{TN+FP}$$

(4) $$P=\frac{TP}{TP+FP}$$

The balanced accuracy (BA) and the F1-score (F1) can be derived from the sensitivity, specificity, and precision as in Equations (5) and (6):

(5) $$BA=\frac{Se+Sp}{2}$$

(6) $$F1=\frac{2\;PSe}{P+Se}$$

For the multiclass classification, given a confusion matrix C, the overall accuracy can be computed by taking the confusion matrix’s trace and dividing it by the sum of each element of the matrix as in Equation (7):

(7) $$Acc=\frac{\sum_{i=1}^{M}C_{ii}}{\sum_{i=1}^{M}\sum_{j=1}^{M}C_{ij}}=\frac{tr\left(C\right)}{\sum_{i=1}^{M}\sum_{j=1}^{M}C_{ij}}=\frac{tr\left(C\right)}{tr\left(C\right)+\sum_{i=1}^{M}\sum_{j=1,i\neq j}^{M}C_{ij}}.$$

In Equation (7), C_ii_ represents the ith element on the confusion matrix’s diagonal, C_ij_ is the element of the confusion matrix on the ith row and the jth column, tr(C) is the trace of the confusion matrix C, and M is the number of classes. The micro-averaging approach was adopted to calculate the sensitivity, specificity, precision, balanced accuracy, and F1-score. In this approach, the sum of TP, TN, FP, and FN across all classes is considered in the definition of the evaluation metrics as described in Equations (8)–(10):

(8) $$Se=\frac{TP}{TP+FN}=\frac{\sum_{k=1}^{M}TP_{k}}{\sum_{k=1}^{M}TP_{k}+\sum_{k=1}^{M}FN_{k}}.$$

(9) $$Sp=\frac{TN}{TN+FP}=\frac{\sum_{k=1}^{M}TN_{k}}{\sum_{k=1}^{M}TN_{k}+\sum_{k=1}^{M}FP_{k}}.$$

(10) $$P=\frac{TP}{TP+FP}=\frac{\sum_{k=1}^{M}TP_{k}}{\sum_{k=1}^{M}TP_{k}+\sum_{k=1}^{M}FP_{k}}.$$

In Equations (8)–(10), *M* represents the total number of classes. Once the sensitivity, specificity, and precision are computed, the balanced accuracy and the F1-score can be computed as defined in Equations (5) and (6).

### 2.6. Description of the Mode of Operation of the Employed Machine Learning Models

This section aims to briefly describe the mode of operation employed for the PCG signal classifications, i.e., SVMs, k-NN, and NNs.

#### 2.6.1. Support Vector Machines

The SVM positions the feature vectors in a space with a known dimension, drawing a hyperplane with the same dimension to categorize the different classes. This hyperplane constitutes the decision boundary for the model. The SVM aims to maximize the margin (i.e., the distance between the nearest samples of two classes along the decision boundary) to enhance model generalization on new samples. Variants of the model include linear SVMs, which employ a hyperplane, and kernel-based SVMs, which work well with datasets not separable by a hyperplane in the original feature space, which is transformed into a higher dimensional space.

#### 2.6.2. k-Nearest Neighbors

The k-NN is a non-parametric model that allows the retention of all its training samples in memory. The classification identifies the *k* closest samples in the training set using several distance functions. The choice of *k* and the distance function depends on the specific application. Popular choices for the distance function include the *L^p^* norm (Minkowski’s distance), the Hamming distance, the cosine similarity, and other functions such as Mahalanobis’ distance or Chebychev’s distance.

#### 2.6.3. Neural Networks

Lastly, NNs are made of interconnected units (also called nodes or neurons), where each connection has a numeric weight, which determines the strength and the direction of the signal propagation among different nodes. The main categories of NNs are feed-forward NNs, where the connections between nodes occur only in one direction, and recurrent NNs, which can process their outputs as inputs. In particular, the nodes of feed-forward NNs are typically organized in layers so that each node in the current layer receives its input only from the nodes of the immediately preceding layer. These feed-forward NNs can be further categorized into single-layer and multi-layer networks.

## 3. Results

Table 4 shows the trained models that achieved the best performance on the test set for the binary classification (with “Normal” and “Pathologic” classes), highlighting the models’ parameters. A k-NN classifier (named k-NN1-B) outperforms all the other trained models, achieving a 98.7% accuracy. Following this, another k-NN classifier (named k-NN2-B) displays a slightly lower overall accuracy on the test set (96.5%). The third-best-performing trained model is a three-layer feed-forward NN (named NN1-B), which reached 96.0% accuracy. Another NN model (called NN2-B) yields a similar performance compared to the previous one (95.9% accuracy). Another k-NN classifier (named k-NN3-B) performs well on the test set, obtaining 95.5% accuracy, followed by a monolayer feed-forward NN (namely, NN3-B) (93.4% accuracy). Lastly, an SVM (“SVM1-B”) provides 93.3% accuracy. Furthermore, Table 4 reports the precision, sensitivity, specificity, F1-score, and BA of the best-performing models for binary classification; the obtained results are then discussed in Section 4.

**Figure 3:**
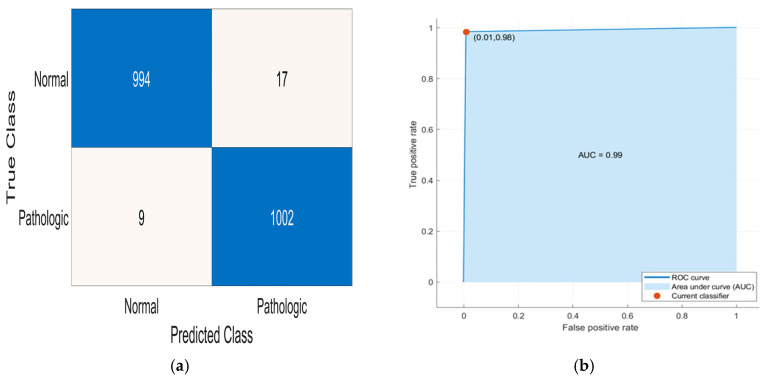

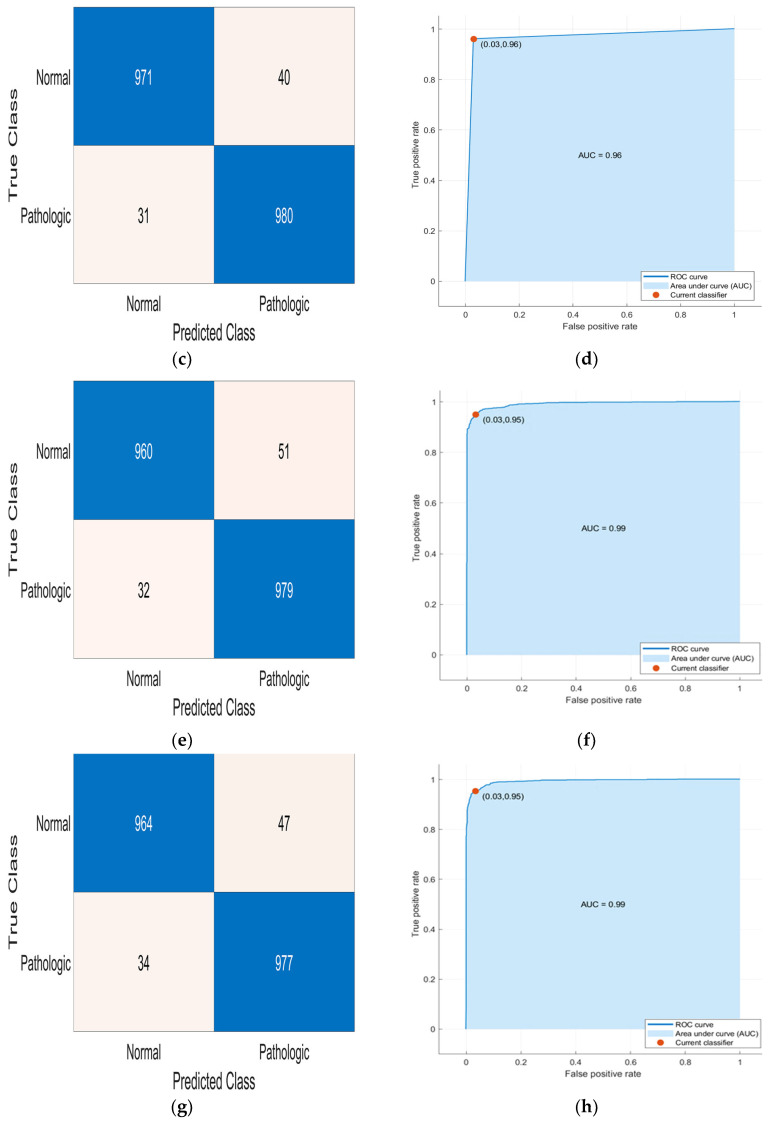

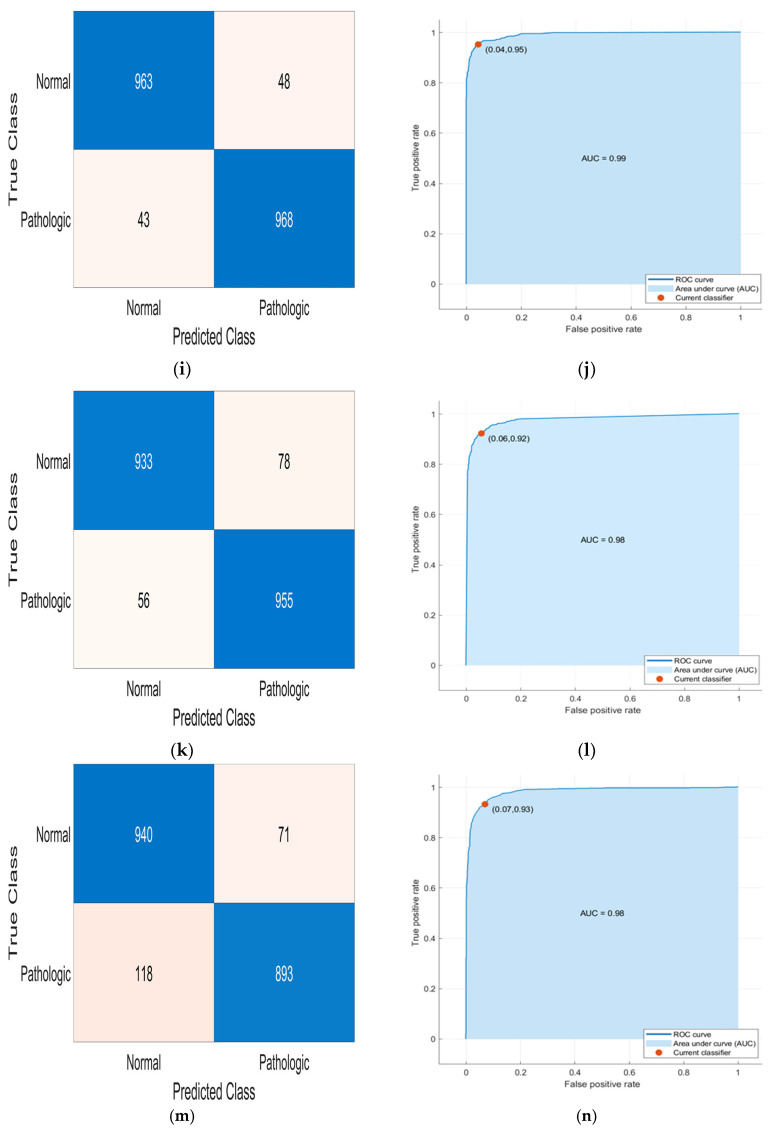
Confusion matrices and ROC curves for best-performing binary PCG classifiers: k-NN1-B (**a**,**b**), k-NN2-B (**c**,**d**), NN1-B (**e**,**f**), NN2-B (**g**,**h**), k-NN3-B (**i**,**j**), NN3-B (**k**,**l**), and SVM1-B (**m**,**n**); the color of each cell of the confusion matrices is representative of the relative numerical value reported; the different colors, associated with the numerical values from the smallest to the highest, can be set in MATLAB when the confusion matrices are printed (for example, from white to dark blue in this case).

Figure 3 shows the confusion matrices and ROC (Receiver Operating Characteristics) curves of the trained models for binary classifications.

**Figure 4:**
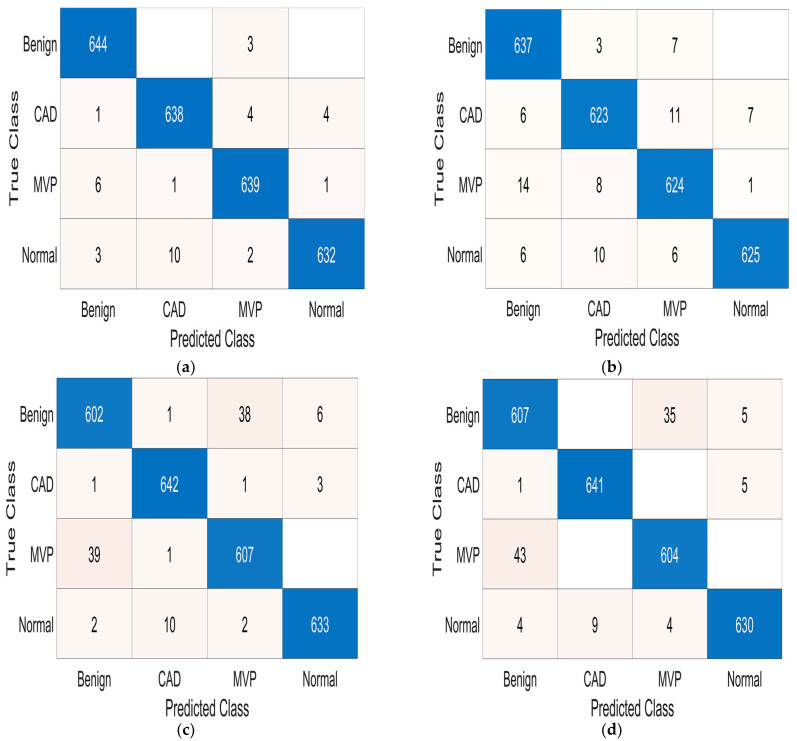

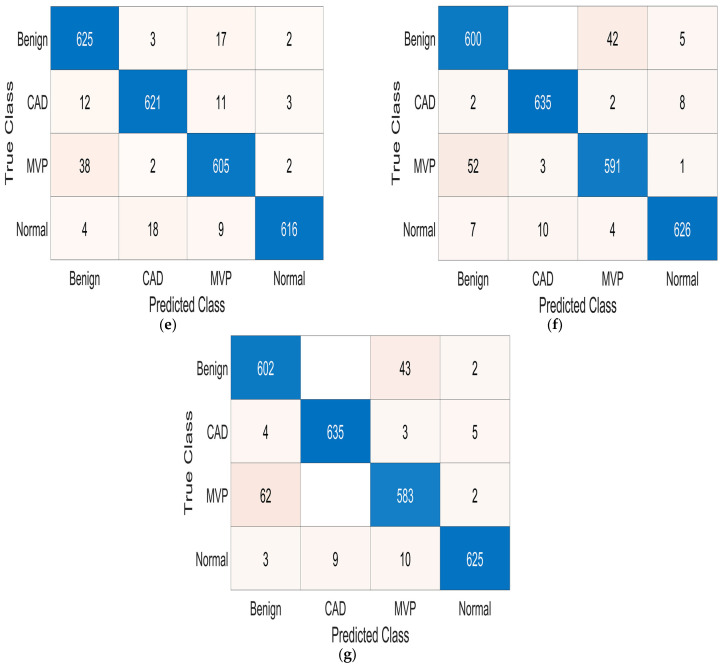
Confusion matrices relative to the best-performing models on the test dataset for the multiclass case: k-NN1-M (**a**), k-NN2-M (**b**), NN1-M (**c**), NN2-M (**d**), k-NN3-M (**e**), NN3-M (**f**), and SVM1-M (**g**); the color of each cell of the confusion matrices is representative of the relative numerical value reported; the different colors, associated with the numerical values from the smallest to the highest, can be set in MATLAB when the confusion matrices are printed (for example, from white to dark blue in this case). Empty white cells in the confusion matrices mean a number of specific cases equal to zero.

Similarly, Table 5 reports the best-performing models trained for multiclass classification. Also in this case, a k-NN classifier (named k-NN1-M), with one neighbor and city block distance, outperforms all other trained models, yielding a 98.6% accuracy on the test set. Following this, another k-NN classifier (named k-NN2-M) achieves slightly lower accuracy than the previous one (96.9%). Also, a trilayered feed-forward NN (called NN1-M) with (200, 100, 50) neurons reaches good performance (96.0% accuracy), followed by another trilayered feed-forward NN classifier (named NN2-M) with (200, 50, 50) neurons, which achieves similar accuracy (95.8%). The next-best-performing trained model is a k-NN classifier (named k-NN3-M) with 10 neighbors and Euclidean distance, which yielded a 95.3% accuracy. Following this, a trilayered feed-forward NN (called NN3-M) with (100, 50, 25) neurons obtains 94.7% accuracy. Finally, an SVM model (called SVM1-M) using a cubic kernel function and a single-level block constraint achieves 94.5% accuracy. Moreover, Table 5 reports the precision, sensitivity, specificity, F1-score, and BA of the best-performing models for the multiclass classification, which are then discussed in Section 4. Figure 4 depicts the confusion matrices of the best-performing multiclass classifiers. The binary and multiclass models exported from MATLAB Classification Learner have been attached to the Supplementary Materials (File S2).

The implemented framing operation with overlap was fundamental for improving the performance of the developed classifiers since they were trained and tested on wider datasets compared to the case without frame overlap. Indeed, in our previous tests involving a smaller dataset for binary classification (2516 frames, equal to about ¼ of the current dataset) obtained by non-overlapping framing with a 5 s window, the best classifier was a k-NN model, reaching 92.9% accuracy (5.8% less than the previous case with a larger dataset). Furthermore, the training and test sets were created by dividing the signals according to the ratio 80%/20% and then framing them using a 5 s window and 1 s time shift (i.e., 4 s overlap). The related balanced training and test sets were constituted by 8084 and 2020 frames (10,104 in total), respectively, as in the dataset obtained by the first method described in Section 2, but without frames relating to the same signal belonging to both training and test sets. Using this methodology, the best-performing classifier was still a k-NN, achieving a 96.4% accuracy on the test set, 2.3% lower than the best classifier obtained using the dataset without imposing the partition of training and test sets at the signal level. In summary, a mean performance reduction of 2.4–2.5% in terms of accuracy was verified for all tested classifiers reported in Table 4, keeping the same ranking obtained with the previous dataset.

Given the procedure used to create the training and test datasets after the signals’ framing, however, partially overlapping frames or non-overlapping frames related to the same original signal might be present in the training and test sets, leading to overestimation of the classifiers’ performance. For this reason, as described in Section 2, we created new balanced training and test datasets, imposing that frames belonging to the same signal cannot be present in both the training and test sets by initially splitting the signals into the two above-mentioned datasets and then performing the framing. Using this dataset for binary classification, for example in the case of no time overlap between frames, the best-performing classifier, namely a k-NN, achieves 90.5% accuracy, 2.4% lower than the best classifier tested with the previous dataset (as reported above).

## 4. Discussion

From the results presented in Section 3, the trained binary classifiers achieve accuracies ranging from 93.3% to 98.7%, which the k-NN1-B model obtains. Table 4 and Table 5 report the performance of the binary and multiclass classifiers in terms of sensitivity, specificity, precision, balanced accuracy, and F1-score. The reported binary and multiclass classifiers perform excellently compared to similar work reported in the scientific literature, as discussed later. These results can be justified by the careful work carried out to construct wide and balanced datasets, exploiting data augmentation techniques. In addition, the parameter optimization of the trained models was a fundamental step in improving their performance. The potential of data augmentation techniques to extend the size of a dataset for training models for the classification of PCG signals has already been demonstrated in [21], allowing the performance of classification models to be improved.

Regarding binary classification, the best-performing classifier is the k-NN1-B model, achieving 98.7% accuracy, precision, F1-score, and balanced accuracy, 98.3 % sensitivity, and 99.1% specificity,. The k-NN algorithm lends itself well to classification problems, demonstrating simplicity and efficiency. Recent studies and practical applications have shown that k-NN can outperform other classification algorithms for PCG classification in specific scenarios [34,35]. Indeed, PCG signals, when transformed into features such as MFCC, might create a feature space where the decision boundaries are relatively simple [35]. Furthermore, k-NN makes decisions based on the local neighborhood of data points, which can be effective if similar PCG signals cluster together in the feature space [34]. In addition, neural networks demonstrated high performance, reaching 96.0% (NN1-B) and 95.9% (NN2-B) accuracy in binary classification. Indeed, NNs can model complex, non-linear relationships in the data, making them suitable for distinguishing between normal and pathological heart sounds that may have subtle differences [36].

A comparison of the best-performing binary classifiers in terms of the area under the curve (AUC), extracted from the ROC curves in Figure 3, and memory occupation of models exported from the MATLAB Classification Learner is reported in Table 6. Specifically, the AUCs of all the considered models exceed 0.96, reaching 0.99 for the best-performing k-NN (k-NN1-B and k-NN3-B) and NN (NN1-B and NN2-B) classifiers, indicating the classifiers’ excellent performance. On the other hand, memory occupation is another important characteristic to be considered for the considered models since they should be implemented on resource-limited platforms. The trained binary classifiers occupy from 92.7 kB (NN3-B) to 11.1 MB (k-NN1-B). In detail, although the k-NN1-B model offers the best performance, it features the largest memory occupation, which is, nevertheless, compatible with its implementation on traditional prototyping platforms (e.g., Raspberry Pi4 (Raspberry Pi Inc., Cambridge, UK), NVIDIA Jetson Nano (NVIDIA Inc., Santa Clara, CA, USA), BeagleBone Black (BeagleBoard Inc., Mansfield, TX, USA), etc.) (Table 6). However, it is necessary to evaluate the performance of the classifiers in relation to the memory occupation to establish the best trade-off between the two characteristics. To this end, the trained binary classifier that offers the best trade-off between performance and memory occupation is NN2-B, which achieves 95.9% accuracy and occupies only 232 kB of memory, enabling its implementation on microcontrollers, like nRF52840 (manufactured by Nordic Semiconductors, Trondheim, Norway), STM32F205 (manufactured by STMicroelectronics, Geneva, Switzerland), or MAX32570 (manufactured by Analog Devices, Norwood, MA, USA).

Relative to the multiclass classification, the trained and test classifiers reach an accuracy ranging from 94.5% to 98.6%, suggesting the achievement of excellent performances on the set of classifiers considered. Similarly to the binary classification, the best performance in terms of accuracy is achieved by a k-NN classifier (k-NN1-M), yielding a 98.6% accuracy. Furthermore, the k-NN1-M model provides the best performance from the point of view of the other metrics (98.6% precision, 98.6% sensitivity, 99.5% specificity, 98.6% F1-score, and 99.1% BA). The reasons for the superior performance of the k-NN algorithm compared to other algorithms have already been explained at the beginning of this section. Also, the non-parametric nature of the k-NN is beneficial in this case, as it does not assume any particular underlying data distribution. This observation can be helpful when the relationship between features and labels is complex and not easily modeled by parametric methods. Similarly to the binary case, even in multiclass classification, the neural networks (NN1-M and NN2-M) obtain excellent performances (96.0% and 95.8% accuracy), close to those obtained by the k-NN1-B algorithm, in agreement with their potential as previously described.

Table 7 below summarizes the AUCs for all classes and the memory occupation of the best-performing trained multiclass models. From the AUCs’ perspective, all multiclass classifiers obtain a one-vs.-all (OvA) AUC greater than or equal to 0.98 across the different classes, indicating a very high ability to rank the classes correctly. The trained multiclass models’ memory occupation spans from 233 kB (NN3-M) to 14.1 MB (k-NN1-M). As for the binary classification’s case, two k-NN-based classifiers (k-NN1-M and k-NN2-M) obtain the best overall performance; however, these models occupy the biggest portion of memory compared to other trained multiclass models (14.1 and 7.19 MB). Still, all the trained multiclass classifiers can be implemented on the same prototyping platforms reported for the binary case (i.e., NVIDIA Jetson Nano, Raspberry Pi 4, and BeagleBone Black). The NN2-M classifier balances performance (95.8% accuracy) and memory occupation (453 kB), allowing for its implementation on microcontroller-based systems such as the already mentioned nRF52840, STM32F205, or MAX32570, as well as on devices based on the STM32U58 (STMicroelectronics, Geneva, Switzerland) microcontroller.

As reported in Section 2, a second method was implemented to build the dataset, imposing that frames related to the same signal cannot belong both to training and test sets. This choice was evaluated to determine the classifiers’ performance in a more realistic scenario where the signals to be classified are different from those used for the training. The two methods, equally used in the scientific literature for dataset creation, were implemented and tested, and the corresponding classifiers’ performance was compared in Section 3. Considering a broad dataset (10,104 frames), the second method led to a mean performance reduction of 2.4–2.5% in terms of accuracy for all the tested classifiers while maintaining the same rank of the classifiers’ performance. Testing both the dataset-creation methods can be considered an added value for the proposed research work, in which we tested and compared the performance of the different classifiers using both methods, providing an indication of how much the performance decreases on average when the second method is used.

Below, Table 8 shows a comparative analysis between classifiers reported in this work and those analyzed in the literature. The main comparison metrics are the overall accuracy, sensitivity, specificity, balanced accuracy, use of heart sound segmentation algorithms, used dataset, employed pre-processing, and whether binary or multiclass classification is performed.

The classifiers trained and tested in this work are computationally simple (e.g., SVMs, feed-forward NNs, and k-NN) and show a good balance between performance and complexity since satisfying metrics are achieved. Other classifiers in the literature that achieved similar performance either use segmentation algorithms, more advanced classification models, or both. This result implies that those models are more resource-intensive than those proposed in this work. For example, P. Krishnan et al. [10] employed a CNN-based classifier, and H. Tang et al. and T. Liu et al. [13,18] used the segmentation algorithm presented in [23], which requires state sequences computed from parameters derived from the ECG signals, such as the R-peak duration and the T wave end time. Moreover, this segmentation algorithm involves logistic regression to estimate the probability of emission and a modified version of the Viterbi algorithm to decode the most likely state sequence. C. Potes et al. [17] employed an AdaBoost and CNN-based classifier using the same segmentation algorithm. In the multiclass case, Y. Zeinali et al. [19] used complex models (i.e., GBC) and segmentation algorithms (i.e., clustering algorithms to identify “S3” and “S4”). Although the classifier proposed by S.B. Shuvo et al. achieves slightly higher performance than our NN2-M (3.8% higher than NN2-M) over a GitHub dataset, it is based on a more complex model (CNN vs. feed-forward NN) and occupies more memory (7.96 MB) than the NN2-M model (435 kB) [20]. All the trained and tested classifiers can be implemented on edge computing wearable devices based on resource-limited platforms such as the Raspberry Pi 4 or the NVIDIA Jetson Nano. These devices running the classifiers developed in this research allow for real-time monitoring and diagnostics of the heart’s condition. In addition, all developed NNs can be implemented on microcontroller-based systems (e.g., STM32F205 or nRF52840), allowing for rapid deployment and scalability of heart diagnostic wearable devices.

Since a denoising step is performed in the pre-processing phase, the classifiers’ performance slightly depends on the unprocessed signal quality, considering that the denoising step’s efficacy cannot perfectly remove all the noises and disturbances. In fact, signal-to-noise ratio (SNR) measurements were carried out before and after applying the denoising, indicating an SNR improvement (i.e., $10\;\log\left(\frac{SNR_{OUT}}{SNR_{IN}}\right)$, where $SNR_{IN}$ and $SNR_{OUT}$ are the signal-to-noise ratio in input and output to the denoising block) spanning from 15 to 30 dB. The classifiers were also trained for the multiclass classification of different heart diseases, allowing for enhanced versatility in different diagnostic scenarios.

## 5. Conclusions

In this research work, different classifier models (i.e., SVM, k-NN, and neural networks) were trained and tested to classify pre-processed PCG signals for both binary (“Normal”/”Pathologic”) and multiclass classifications (“Normal”/”CAD”/”MVP”/”Benign”). Two datasets of 482 and 826 signals extracted from the Physionet/CinC 2016 database were constituted to train and test the binary and multiclass models, respectively. A pre-processing chain was realized to remove spikes, reduce noise, and normalize the PCG signals, split into 5 s frames using a 1 s shift. Then, a feature set was extracted from the PCG frames and used to train and test the classifiers. The test results demonstrated that the k-NN-based models obtained the best performance for both binary and multiclass classification, reaching 98.7% accuracy and F1-score, but requiring a large amount of memory occupation (up to 11.1 and 14.1 MB for binary and multiclass classification). Nevertheless, NN-based models obtained a good trade-off between performance and memory occupation, obtaining up to 96.0% accuracy and low memory occupation (≤735 kB), thus enabling their implementation on resource-limited systems. In the future, the trained and tested classifiers could be implemented in edge computing-based systems or mobile healthcare applications, allowing for real-time diagnosis of the heart’s abnormalities or specific diseases in the case of multiclass classifiers. Then, the effectiveness of the denoising step was analyzed, demonstrating an improvement in the SNR in the range from 15 to 30 dB. Therefore, the developed classifiers can be deployed for low-quality PCG signals and recorded in noisy environments. As a future step, the multiclass classification could be improved by optimizing the features extracted for each disease. Also, the SNR of misclassified frames could be analyzed to gain insights into the relationship between performance and signal quality.

## Figures and Tables

**Table 1:** Types of data in each database of the Physionet/CinC 2016 dataset’s training set.

Database	PCG Signal Labels in the Dataset
“training-a”	“Normal”, “MVP” (mitral valve prolapse), “AD” (Aortic Disease), “MPC” (Miscellaneous Pathological Conditions)
“training-b”	“Normal”, “CAD” (coronary artery disease)
“training-c”	“Normal”, “MR” (Mitral Regurgitation), “AS”(Aortic Stenosis)
“training-d”	“Normal: NHC” (signals acquired from 19 subjects aged between 18 and 40), “Normal: MARS500”(signals acquired from 6 volunteer astronauts), “Pathologic”
“training-e”	“Normal”, “CAD”
“training-f”	“Normal”, “Pathologic”

**Table 2:** Optimization of the wavelet denoising parameters.

Wavelet	Order	Level	$\bar{\mathrm{SNR}}$ [dB]
Daubechies (dB)	4	6	21.6
Daubechies (dB)	4	8	20.5
Daubechies (dB)	4	10	20.3
Daubechies (dB)	5	6	23.4
Daubechies (dB)	5	8	23.1
Daubechies (dB)	5	10	22.4
Symlet (sym)	6	6	17.6
Symlet (sym)	6	8	17.4
Symlet (sym)	6	10	16.9
Symlet (sym)	10	6	15.4
Symlet (sym)	10	8	15.7
Symlet (sym)	10	10	14.9

**Table 3:** Extracted features and their respective domains.

Domain	Extracted Feature	Definition
Time	Signal (Shannon) entropy	$-\sum_{i=1}^{N}p\left(x_{i}\right)\left(p\left(x_{i}\right)\right)$, where p(x_i_) represents the occurrence probability of each signal component, and N is the signal length.
	Heart rate	The heart cycle duration is the time from lag zero to the highest peak within 500–2000 ms in the signal’s homomorphic envelope autocorrelation [22]. The heart rate is obtained by dividing this duration by 60. The systolic interval is the time between the lag zero and highest peak occurring between 200 ms and half of the heart cycle [22].
	Systolic time interval	
Frequency	Maximum power spectrum amplitude	$max{\left|DFT\left(x\right)\right|}^{2}$; in this work the mono-lateral DFT (discrete Fourier transform) is considered.
	Dominant frequency	${argmax}_{f}{\left|DFT\left(x\right)\right|}^{2}$
	Maximum spectrum ratio	The ratio between the maximum power spectrum and the sum of the overall power spectrum
	Normalized spectral entropy	The normalized spectral entropy is calculated by dividing the power spectrum by its cumulative sum, determining the Shannon entropy and dividing it by ${log}_{2}N$.
Statistical	Mean	$\bar{x}=\frac{1}{N}\sum_{i=1}^{N}x_{i}$
	Median	The 50th percentile (second quartile)
	Standard deviation (SD)	$\sigma =\sqrt{\frac{1}{N-1}\sum_{i=1}^{N}{\left(x_{i}-\bar{x}\right)}^{2}}$
	Mean absolute deviation (MAD)	$\frac{1}{N}\sum_{i=1}^{N}\left|x_{i}-\bar{x}\right|$
	First quartile (Q1)	The 25th percentile
	Third quartile (Q3)	The 75th percentile
	Interquartile range (IQR)	Q3 − Q1
	Skewness	$\frac{N}{\left(N-1\right)\left(N-2\right)}\sum_{i=1}^{N}{\left(\frac{x_{i}-\bar{x}}{\sigma }\right)}^{3}$
	Kurtosis	$\frac{N(N+1)}{\left(N-1\right)\left(N-2\right)(N-3)}\sum_{i=1}^{N}{\left(\frac{x_{i\;}-\bar{x}}{\sigma }\right)}^{4}-\frac{N{\left(N-1\right)}^{2}}{\left(N-2\right)\left(N-3\right)}$
Wavelet	Wavelet entropy for the approximation coefficients at level 4	The wavelet entropy is obtained by computing the Shannon entropy of the normalized energy of wavelet coefficients at each decomposition level.
	Wavelet entropy for the detail coefficients at level 4	
	Wavelet entropy for the detail coefficients at level 3	
	Wavelet entropy for the detail coefficients at level 2	
MFCCs	1st of the MFCCs	MFCCs are extracted as follows: The signal is windowed using the Hamming window, followed by the fast Fourier transform (FFT). The resulting spectrum is filtered through Mel-scale triangular filters, with the relation between the Mel scale and frequency given by $Mel\left(f\right)=1127\;log\left(1+\frac{f}{700}\right)$. Finally, the discrete cosine transform (DCT) is performed on the Mel spectrogram to obtain the MFCCs [30].
	2nd of the MFCCs	
	3rd of the MFCCs	
	4th of the MFCCs	
	5th of the MFCCs	
	6th of the MFCCs	
	7th of the MFCCs	
	8th of the MFCCs	
	9th of the MFCCs	
	10th of the MFCCs	
	11th of the MFCCs	
	12th of the MFCCs	
	13th of the MFCCs	

**Table 4:** Performance of the best-performing models for the binary classification.

Model	Accuracy [%]	Precision [%]	Sensitivity [%]	Specificity [%]	F1-Score [%]	BA [%]	Parameters
k-NN1-B	98.7	98.7	98.3	99.1	98.7	98.7	Number of neighbors: 1 Distance metric: city block Distance weight: inverse Standardized data: yes
k-NN2-B	96.5	96.9	96.0	96.9	96.4	96.5	Number of neighbors: 1 Distance metric: Euclidean block Distance weight: equal Standardized data: yes
NN1-B	96.0	96.8	95.0	96.8	95.9	95.9	Trilayered: (200, 100, 50) neurons Activation: ReLU λ = 4.1279 × 10^−7^ Iteration limit: 1000 Standardized data: yes
NN2-B	95.9	96.6	95.4	96.6	96.0	96.0	Trilayered: (100, 50, 25) neurons Activation: ReLU λ = 4.1279 × 10^−7^ Iteration limit: 1000 Standardized data: yes
k-NN3-B	95.5	95.7	95.3	95.7	95.5	95.5	Number of neighbors: 10 Distance metric: Euclidean Distance weight: squared inverse Standardized data: yes
NN3-B	93.4	94.3	92.3	94.5	93.3	93.4	Single layer: 100 neurons Activation: ReLU λ = 0 Iteration limit: 1000 Standardized data: yes
SVM1-B	93.3	88.8	93.0	88.3	90.9	90.7	Kernel function: cubic Kernel scale: automatic Box constraint level: 1 Standardized data: yes

**Table 5:** Performance of the best-performing models for the multiclass classification.

Model	Accuracy [%]	Precision [%]	Sensitivity [%]	Specificity [%]	F1-Score [%]	BA [%]	Parameters
k-NN1-M	98.6	98.6	98.6	99.5	98.6	99.1	Number of neighbors: 1 Distance metric: city block Distance: squared inverse Standardized data: yes
k-NN2-M	96.9	96.9	96.9	99.0	96.9	98.0	Number of neighbors: 1 Distance metric: Euclidean block Distance weight: equal Standardized data: yes
NN1-M	96.0	96.0	96.0	98.7	96.0	97.4	Trilayered: (200, 100, 50) neurons Activation: ReLU λ = 1.612 × 10^−7^ Iteration limit: 1000 Standardized data: yes
NN2-M	95.8	95.9	95.9	98.6	95.9	97.3	Trilayered: (200, 50, 50) neurons Activation: ReLU λ = 0 Iteration limit: 1000 Standardized data: yes
k-NN3-M	95.3	95.3	95.3	98.4	95.3	96.9	Number of neighbors: 10 Distance metric: Euclidean Distance weight: squared inverse Standardized data: yes
NN3-M	94.7	94.7	94.7	98.2	94.7	96.5	Trilayered: (100, 50, 25) neurons Activation: ReLU λ = 1.612 × 10^−7^ Iteration limit: 1000 Standardized data: yes
SVM1-M	94.5	94.5	94.5	98.2	94.5	96.4	Kernel function: cubic Kernel scale: automatic Box constraint level: 1 Standardized data: yes

**Table 6:** Summary table with the AUC and the memory occupation of the best-performing models for binary PCG classification.

Model	AUC	Memory Occupation
k-NN1-B	0.99	11.1 MB
k-NN2-B	0.96	5.67 MB
NN1-B	0.99	734 kB
NN2-B	0.99	232 kB
k-NN3-B	0.99	5.67 MB
NN3-B	0.98	92.7 kB
SVM1-B	0.98	1.26 kB

**Table 7:** Summary table with the AUCs for each class and the memory occupation of the tested multiclass classifiers.

Model	AUC	Memory Occupation
k-NN1-M	1.00 (Benign), 0.99 (CAD), 0.99 (MVP), 0.99 (Normal)	14.1 MB
k-NN2-M	0.99 (Benign), 0.98 (CAD), 0.98 (MVP), 0.98 (Normal)	7.19 MB
NN1-M	0.99 (Benign), 1.00 (CAD), 0.99 (MVP), 1.00 (Normal)	735 kB
NN2-M	0.99 (Benign), 1.00 (CAD), 0.99 (MVP), 1.00 (Normal)	453 kB
k-NN3-M	1.00 (Benign), 1.00 (CAD), 1.00 (MVP), 0.99 (Normal)	7.19 MB
NN3-M	0.99 (Benign), 1.00 (CAD), 0.99 (MVP), 1.00 (Normal)	233 kB
SVM1-M	0.99 (Benign), 1.00 (CAD), 0.98 (MVP), 1.00 (Normal)	2.82 MB

**Table 8:** Comparative analysis between the classifiers proposed in the literature and the ones trained and tested in this work.

Reference	Classifier Type	Acc [%]	Se [%]	Sp [%]	BA [%]	Heart Sound Segmentation Algorithms	Dataset Used for Classifiers’ Training	Pre-Processing	Binary/Multiclass Classification
P. Langley et al. [8]	Threshold classifier	N.A. (*)	98	54	77	No	Physionet/CinC 2016	Only the first 5 s of signals considered	Binary (Normal/Pathological)
P. Langley et al. [9]	Classification tree	N.A. (*)	77	80	79	No	Physionet/CinC 2016	Reduced noise signal segments considered	Binary (Normal/Pathological)
P. Krishnan et al. [10]	CNN	85.65	86.73	84.75	85.74	No	Physionet/CinC 2016	Resampling, signal division in 5 s frames, Savitzky–Golay filter	Binary (Normal/Pathological)
N. Singh-Miller et al. [11]	RF	N.A. (*)	76	87	81	No	Physionet/CinC 2016	N.A. (*)	Binary (Normal/Pathological)
M.A. Goda et al. [12]	SVM	N.A. (*)	77.2	85.2	81.2	Yes	1000 samples (1:1 Normal/Pathological) from Physionet/CinC 2016 d	Resampling and bandpass filtering	Binary (Normal/Pathological)
H. Tang et al. [13]	SVM	N.A. (*)	88	87	88	Yes	Physionet/CinC 2016	Highpass filtering, spike removal, and normalization	Binary (Normal/Pathological)
T. Nilanon et al. [14]	CNN	N.A. (*)	77	85.3	81.1	N.A. (*)	Physionet/CinC 2016	Division of the signal into 5 s frames	Binary (Normal/Pathological)
M. Homsi et al. [15]	Ensemble Classifier	N.A. (*)	79.60	80.60	80.10	Yes	Physionet/CinC 2016	Resampling	Binary (Normal/Pathological)
E. Kay et al. [16]	DropCon-nected NN	N.A. (*)	N.A. (*)	N.A. (*)	85.20	Yes	Physionet/CinC 2016	No	Binary (Normal/Pathological)
C. Potes et al. [17]	AdaBoost and CNN	N.A. (*)	94.24	77.81	86.02	Yes	Physionet/CinC 2016	Resampling, bandpass filtering, spike removal	Binary (Normal/Pathological)
T. Liu et al. [18]	SVM	90.9	87.8	93.0	90.4	Yes	Custom dataset of 991 samples	Highpass filter, 50 Hz notch filter, division in 5 s segments	Binary (Normal/CAD)
Y. Zeinaili et al. [19]	GBC	87.5	87.5	93.75	90.63	Yes	156 PCG signals from the PASCAL dataset	Savitzky–Golay filter	Multiclass (Normal /Abnormal S3/Abnormal S4)
S.B. Shuvo et al. [20]	CRNN	86.6	90.3	N.A. (*)	N.A. (*)	No	Physionet/CinC 2016	Resampling and normalization	Binary (Normal /Pathological)
N. Baghel et al. [21]	CNN	98.6	N.A. (*)	N.A. (*)	N.A. (*)	N.A. (*)	2000 samples from data from GitHub	Bandpass filtering	Multiclass (Normal/MS/AS/MR/MVP)
This work	k-NN (k-NN1-B)	98.7	98.3	99.1	98.7	No	10,104 samples (1:1 Normal/Pathologic) from 482 signals of Physionet/CinC 2016	Spike removal, denoising, bandpass filter, normalization, signal division in 5 s frames with 1 s shift	Binary (Normal/Pathological)
	Feed-forward NN (NN2-B)	95.9	95.4	96.6	96.0	No	10,104 samples (1:1 Normal/Pathologic) from 482 signals of Physionet/CinC 2016	Spike removal, denoising, bandpass filter, normalization, signal division in 5 s frames with 1 s shift	Binary (Normal/Pathological)
	k-NN (k-NN1-M)	98.6	98.6	99.5	99.1	No	13,136 samples (1:1:1:1 Normal/CAD/MVP/Benign) from 826 signals of Physionet/CinC 2016	Spike removal, denoising, normalization, signal division in 5 s frames with 1 s frameshift	Multiclass (Normal/CAD/MVP/Benign)
	Feed-forward NN (NN2-M)	95.8	95.9	98.6	97.3	No	13,136 samples (1:1:1:1 Normal/CAD/MVP /Benign) from 826 signals of Physionet/CinC 2016	Spike removal, denoising, normalization, signal division in 5 s frames with 1 s shift	Multiclass (Normal/CAD/MVP/Benign)

(*) N.A.: Not available.

## Data Availability

The data are available upon request.

## Supplementary Materials

The following supporting information can be downloaded at https://www.mdpi.com/article/10.3390/s24123853/s1: File S1: Datasets and annotation files for the binary and multiclass classifiers. File S2: Tested binary and multiclass MATLAB models.
